# Testicular tissue cryopreservation and transplantation as a strategy for feline conservation: a review of research advances

**DOI:** 10.3389/fvets.2025.1572150

**Published:** 2025-04-02

**Authors:** Zhiqiang Han, Xin Liu, Haijun Wang, Izhar Hyder Qazi, Luyao Wang, Rui Du, Xiangpeng Dai, Chao Xu

**Affiliations:** ^1^College of Animal Science and Technology, Jilin Agricultural University, Changchun, China; ^2^Institute of Zoology, Chinese Academy of Sciences, Beijing, China; ^3^Jilin Province Northeast Tiger Garden and Jilin Wild Animal Rescue Breeding Center Committee, Changchun, China; ^4^Guangdong Provincial Key Lab of Agro-Animal Genomics and Molecular Breeding, College of Animal Science, South China Agricultural University, Guangzhou, Guangdong, China; ^5^Shaheed Benazir Bhutto University of Veterinary and Animal Sciences, Sakrand, Pakistan; ^6^Key Laboratory of Organ Regeneration and Transplantation of Ministry of Education, First Hospital of Jilin University, Changchun, China; ^7^National-Local Joint Engineering Laboratory of Animal Models for Human Disease, First Hospital, Jilin University, Changchun, China

**Keywords:** big cats, fertility, preservation, sperm, vitrification

## Abstract

As we humans continue our detrimental activities on the planet, the biodiversity loss is now seen as a big threat to entire ecosystem in which we all live. This issue becomes even more critical as we see a rapid increase in the number of animal species being listed as endangered, and a far greater rate of species extinction. We all know that felines play a crucial part in our ecosystems, it is therefore safe to argue that their conservation could play an important role in minimizing the biodiversity loss. Advanced reproductive biotechnologies including testicular tissue cryopreservation and transplantation are considered as effective tools for the conservation of animal species. As we have seen with the Giant Panda, these biotechnologies could offer new possibilities for the conservation of other endangered species including felines. Although previously a few wild feline spp. were conserved by this method, little is known about the factors influencing the efficiency of these methods. Therefore, if we are to maximize the conservation efforts, further optimization of these biotechnologies is required to achieve better conservation results. In this article, we present an overview of testicular tissue of felines and the factors influencing testicular tissue cryopreservation and testicular graft recovery in felines.

## 1 Introduction

In recent years, we have seen a significant rise in the global biodiversity crisis which is undoubtedly accelerated by the direct or indirect human activities. It is said that this rapid loss of biodiversity might trigger the sixth mass extinction crisis (1, 2). According to the International Union for Conservation of Nature (IUCN), a great proportion, 75% of felines are now classified as threatened or endangered species. Notably, the population of big cats is significantly declining and given that they sit at the top of the food chain, this decline is seen as one of the factors contributing to the biodiversity crisis (3, 4). The wildlife conservation not only helps in maintaining the effective population size but it is equally important for ensuring the genetic diversity. Fortunately, the preservation of biological samples in biobank have played a crucial role in reinstating the population of rare and endangered species (5, 6). Sperm cryopreservation is one of the key strategies for the preservation of male genetic resources. However, the collection of semen samples from wild species, particularly endangered species, comes with inherent challenges of the complexity, accessibility and availability of these animals.

Testicular tissue cryopreservation has emerged as a promising technique for conserving species diversity (7–9). This method could be particularly valuable in conserving the species whose sperm cannot be obtained due to factors such as the unexpected death, seasonal availability of species, and males with specific pathologies (e.g., azoospermia). The primitive germ cells, spermatogonial stem cells, are the important cell type in the preserved testicular tissue (10). Spermatogonial stem cells are considered as origin of sperm to maintain normal spermatogenesis throughout the reproductive lifespan of most male mammals (11). Importantly, the combination of testicular tissue cryopreservation and transplantation of frozen–thawed testicular tissue has demonstrated the capability to restore fertility in male animals (9). Furthermore, the application of testicular tissue cryopreservation has now been demonstrated in several felines (big cats) species including the jungle cat (*Felis Chaus*), lion (*Panthera Leo*), leopard (*Panthera Pardus*), iberian bobcat (*Lynx pardinus*), and persian leopard (*Panthera pardus saxicolor*) (12–14).

The efficiency of testicular tissue preservation and transplantation methods is influenced by many factors, including the environmental temperature at which the animal died, interval between animal death and specimen collection, transportation conditions, culture medium, age of animal, and type of tissue freezing method, among others. Therefore, if we are to increase the success rate and subsequently improve the conservation of species, the optimization of testicular tissue preservation and transplantation methods should be given due attention. In this article, we present an overview of histological features of feline testicular tissue and discuss the main procedures of testicular tissue preservation and transplantation, and finally highlight the major factors that influence the efficiency of these methods. We hope that this review will provide relevant information for the long-term conservation of genetic resources and biodiversity of felines.

## 2 Histological features of feline testes

Histologically, testicular tissue is composed of supporting structures (*tunica albuginea*), septum, and lobules, tubular structures (seminiferous tubules, ductuli efferentes), and collecting structures (rete testis and epididymis). Feline males usually attain puberty approximately at 8–10 months of age (15). The big cats (*Pantherinae*) including cheetahs (*Acinonyx jubatus*), lions (*Panthera leo*), and tigers (*Panthera tigris*) attain late puberty, typically around 1–2 years of age (16, 17). During puberty, enlargement of the testes is primarily attributed to the seminiferous tubules and the Leydig cells. The length of the seminiferous tubules is increased rather than an expansion in the diameter. The Leydig cells undergo significant transformations throughout development, enhancing their capacity to synthesize testosterone, a hormone crucial for the maturation of genital structures. In mammals, the range of tubular and intertubular ratios falls within 70 to 90%, while the tubular diameter spans from 180 to 300 μm (18, 19).

In general, spermatogenesis and androgen secretion are the two main functions of mammalian testes. The development of feline testes and spermatogenesis may be influenced by several factors such as season, animal body size, testicular structure, and hormonal regulation. Seasonal estrus is a key factor limiting feline testicular development. As seasonal breeders, felines exhibit an increase in testicular size during the breeding season. Similar to domestic cat, males of captive ocelot (*Leopardus pardalis*) and margay (*Leopardus wiedii*) species exhibit year-round spermatogenic activity, with a peak at summer months (20, 21). Feline testes also display considerable variation in size, with testicular volume ranging from 1.53 cm^3^ in tigrina (*Leopardus guttulus*) to 62.43 cm^3^ in jaguar (*Panthera onca*) (19). There is limited species-specific data on feline reproductive physiology, particularly the big cats, therefore, further investigation is required to improve our understanding in this field and to help us better understand their testicular function and maintain the genetic resources of the endangered felines.

## 3 The cryopreservation of feline testicular tissue

### 3.1 Collection of feline testicular tissue

The collection of testicular tissue for cryopreservation is mainly done from wild animals raised in farms, laboratories, breeding centers, zoos, and natural habitats (22). Testicular tissue can be obtained through orchiectomy of mature or prepubertal males, or postmortal from animals who have died in the wildlife due to aging or accidentally (e.g., roadkills) or due to surgical complications, and while sampling of biopsies (23, 24). The collection of feline testicular tissue has been performed on a few species including jungle cat, lion, leopard, the Persian leopard, and the Spanish lynx (12–14). Normally, the prepubertal testis contains a higher proportion of spermatogonial stem cells, and hence is considered as a better resource for tissue preservation. The prepubertal testicular tissue was collected from under six-months old cub of the Spanish lynx, while adult testicular tissue has been collected from the jungle cat, leopard and the Spanish lynx (see Table 1 for further information). The collected feline testis samples are delivered to the laboratory between 9 and 48 h after death, leaving a significant effect on the efficiency of cryopreservation (12–14). Although it is believed that the prepubertal feline testes are suitable for cryopreservation, the limitations of age on the restoration of testicular tissue may be gradually decreased with the advances in biotechnologies.

**Table 1:** Progress in frozen storage and transplantation of testicular tissue in wild (captive) felines.

Animals	Latin name	Age	Cause of death	Death to laboratory (h)	Testicular fragments size	Freezing media	Freezing method		% Recovery		References
									~40 weeks	>40 weeks	
Jungle cat	*Felis chaus*	10 Years	Anesthetic complication	27	3 × 3 × 3 mm	S: 0.1 M sucrose +11% DMSO +5 mL protein free PBS R: 15mLmTCM 199 + 0.5 M sucrose +20% FCS + 15% DMSO +15% ethylene glycol	Slow freezing	Rapid freezing	—	—	(12)
Lion	*Panthera leo*	6 Months	Pericarditis	63	3 × 3 × 3 mm		Slow freezing	Rapid freezing	—	—	
Leopard	*Panthera pardus*	10 years	Aging	9	3 × 3 × 3 mm		Slow freezing	Rapid freezing	—	—	
Persian leopard	*Panthera pardus saxicolor*	12 years	Aging	16	2 × 2 × 2 mm	DMEM F-12 supplemented with 20% FBS and 2.8 M DMSO	Slow freezing		—	—	(13)
Spanish Lynx	*Lynx pardinus*	6 weeks fetus	Maternal stress	36	~1 mm^3^	20% FBS + 60% DMEM +20% DMSO	Slow freezing		0%	0%	(14)
Spanish Lynx	*Lynx pardinus*	1.5 days	Hypothermia	36	~1 mm^3^		Slow freezing		0%	0%	
Spanish Lynx	*Lynx pardinus*	3 days	Unknown	12	~1 mm^3^		Slow freezing		0%	0%	
Spanish Lynx (1)	*Lynx pardinus*	6 months	Road kill	24	~1 mm^3^		Slow freezing		75.7 ± 8.5%	56.3 ± 6.3%	
Spanish Lynx (2)	*Lynx pardinus*	6 months	Road kill	24	~1 mm^3^		Slow freezing		33.30%	33.3 ± 10.5%	
Spanish Lynx	*Lynx pardinus*	2 years	Feline leukemia	48	~1 mm^3^		Slow freezing		0%	0%	

S stands for slow freezing, R stands for rapid freezing.

### 3.2 Cryopreservation of testicular tissue

**Figure 1:**
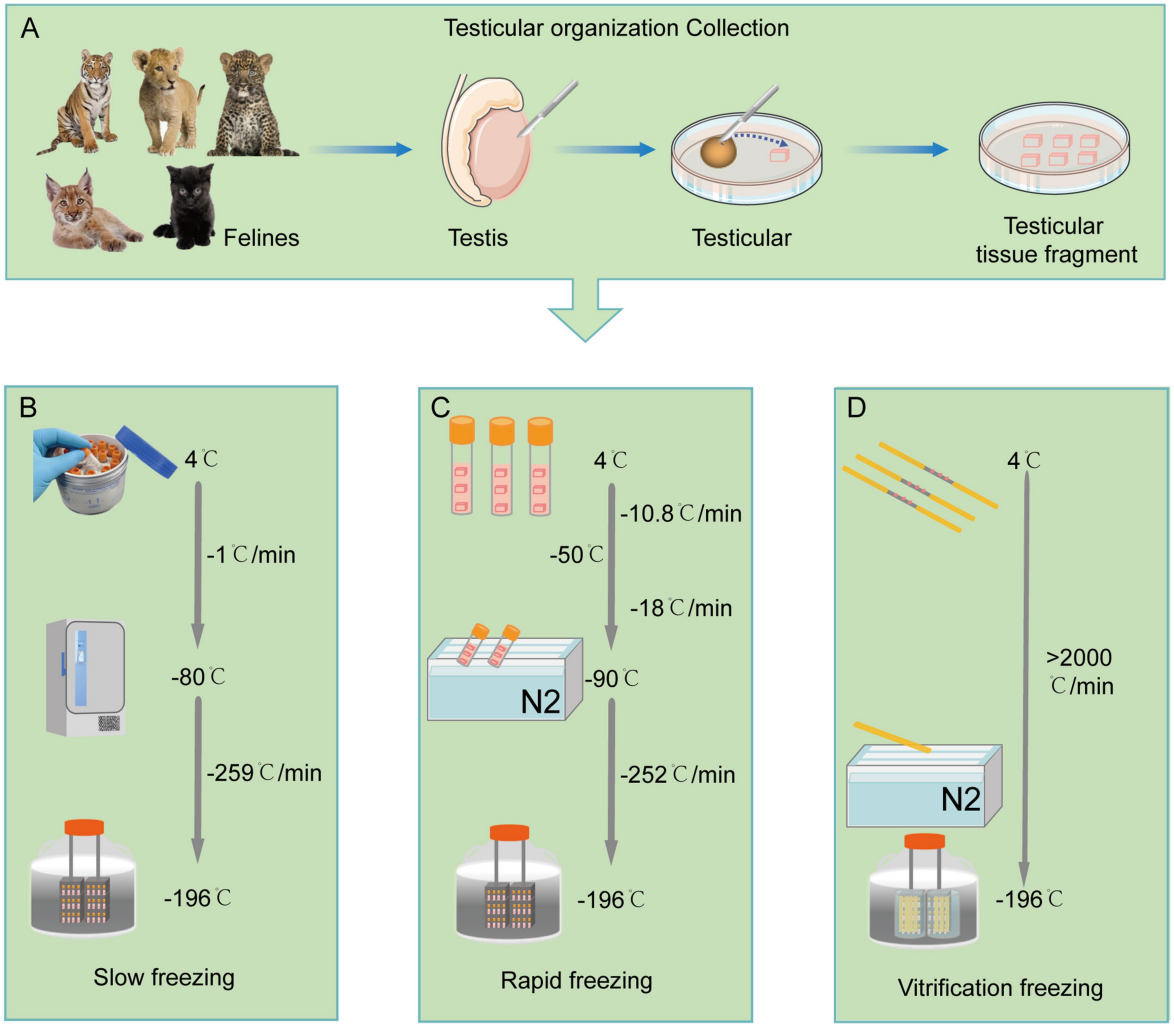
The cryopreservation of felines testicular tissue. **(A)** Collection and processing procedure for felines testicular tissue fragments. **(B)** Slow freezing procedure of feline testicular tissue fragments. **(C)** Rapid freezing procedure of feline testicular tissue fragments. **(D)** Vitrification freezing procedure of feline testicular tissue fragments.

There are two major strategies for the preservation of testicular tissue, short-term preservation at low temperatures and long-term preservation at ultra-low temperatures (22). Cryopreservation is an effective approach for long-term storage. In general, there are three common methods for the cryopreservation of testicular tissue including slow freezing, rapid freezing, and vitrification. These methods are pictorially summarized in Figure 1.

#### 3.2.1 Slow freezing of testicular tissue

Slow freezing is a process of gradual cooling at a rate of −1°C/min within the temperature range of 4°C to −80°C. Subsequently, a rapid cooling rate of −259°C/min is applied between −80°C and −196°C to mitigate potential cytotoxic effects (14). Slow freezing of testicular tissue is a cheaper and more convenient cryopreservation method (8). It has been demonstrated in humans, buffaloes, cows, sheep, cats, pigs, and monkeys that the slow freezing of testicular tissue can preserve the cellular ultrastructure, tubular morphology, and tissue function (8, 25, 26). Slow freezing provides an optimal cooling rate to the cells to reduce the damage caused by the formation of intracellular ice crystal.

#### 3.2.2 Rapid freezing of testicular tissue

In contrast to slow freezing, rapid freezing uses a three-step cooling mode which includes the cooling rate of −10.8°C per minute from 4°C to −50°C, a cooling rate of −18°C per minute from −50°C to −90°C, and a cooling rate of −252°C per minute from −90°C to −196°C (27). Both slow freezing and fast freezing techniques have been used to preserve feline testicular tissue (14). In felines, the effects of fast and slow freezing of testicular tissue were assessed in jungle cats, lions and leopards (14). The testicular tissue subjected to slow freezing exhibited a better membrane integrity and DNA integrity, but compared to slow freezing, the exposure time to the cryoprotective reagent is shorter in fast freezing, minimizing the negative effect (13).

#### 3.2.3 Vitrification of testicular tissue

Vitrification, the ultra-rapid method of cryopreservation, is a cheaper and easily implemented freezing method. However, the reports on application of vitrification in cryopreservation of feline testicular tissue are limited. Vitrification has advantages of preserving the testicular lumen, cellular junctions and cellular membrane integrity, as well as spermatogonial nuclei which is extremely important for the proliferative capacity of them (28). Therefore, due to the ultra-rapid cooling speed, vitrification can reduce the ice crystals in the cell during freezing and maintain the original histomorphology, cell proliferation potential and cellular viability of testicular tissue in prepubertal cats (29). Importantly, recent studies have shown that slow freezing is good at preserving the viability and DNA integrity of elongated cells, whereas vitrification is a better method to maintain the integrity of round testicular cells (29–31). This contrasting evidence calls for further species-specific focused studies to optimize the cryopreservation technique and strategies to achieve a better efficiency for the feline testicular tissue preservation.

### 3.3 The potential factors influencing testicular tissue cryopreservation efficiency

#### 3.3.1 The volume of testicular tissue and cryopreservation efficiency

The size of cryopreserved testicular tissue is an important factor influencing the effectiveness of cryopreservation. Previous studies have shown that feline testicular tissue fragments could be cryopreserved at size of ~1 mm^3^, 8 mm^3^ (2 × 2 × 2 mm), and 27 mm^3^ (3 × 3 × 3 mm). In studies on rats, the testicular tissue fragments of 8–18 mm^3^ (2–3 mm in length, 2–3 mm in width, 2 mm in thickness) were found to be more suitable for cryopreservation and transplantation (32). In humans, the cryopreserved testicular tissue fragments of ∼2–8 mm^3^ was reported to maintain the fertility (33, 34). It was found that too small or thin tissue was easily resorbed during transplantation in rat. Studies on rats have found that 23% of frozen testicular tissues (1 × 1 × 1 mm) develop cellular depletion 30 days after transplantation (32). Moreover, the larger tissue fragments are easy to vascularise and survive from ischaemic injury than the smaller tissue fragments (32). But oversized testicular tissue fragments demonstrated reduced capability in graft recovery, poor vasculature integrity, smaller renal tubule diameter, decreased spermathecal number, and declined microvessel density (35). This evidence indicates that the physical size of the tissue is indeed an important factor that influences the efficiency of a given cryopreservation scheme, which may be related to the distance between the surface of testis and the inner cells, the size of testicular tissue fragments affects the efficiency of cryopreservation solution permeation (32, 35). This is especially true for vitrification, as the sample size is a critical variable for the successful solidification of the tissue aqueous milieu into a non-crystalline glassy phase (8). Based on existing research, we believe that the size of the testicular tissue for cryopreservation in felines should be above 1 mm^3^. But there is need for species-specific studies focusing on optimization of the size of tissue fragment to get best cryopreservation efficiency, as different feline species may require different freezing strategies.

#### 3.3.2 Cryoprotectant agents and cryopreservation efficiency

Cryoprotectants are important reagents that protect the testicular tissue from mechanical damage during cryopreservation. The cryoprotectant can not only reduce the effect of ice crystals formed during the freezing process, maintain osmotic balance, and minimize the cellular dehydration and shrinkage induced by the cooling process, but also reduce cytotoxicity (36). The optimal cryoprotectants can as well maintain the cellular quality of testicular tissue fragments after freezing and thawing. Currently, the membrane-permeable and non-membrane-permeable agents are the two commonly used cryoprotectants. Notably, many types of membrane-permeable cryoprotectants including dimethyl sulfoxide (DMSO), ethylene glycol (EG) and glycerol (GLY) have been tested in cryopreservation of feline genetic resource (37). Interestingly, the addition of some non-permeable cryoprotectants such as sugars can increase the extracellular osmotic pressure and enhance cellular dehydration, thus maintain cell morphology by reducing ice crystal formation (38).

DMSO is the widely used cryoprotectant for the cryopreservation of testicular tissue of various animals, and it is also suitable for preserving testicular tissue from immature animals (39). EG was shown to be suitable for the cryopreservation of testicular tissue from adult animals (40). GLY is a well-tolerated cryoprotectant and was shown to have positive effect on the cryopreservation of pre-pubertal testicular tissue from pre-pubertal animals. These findings have been confirmed in studies on human testicular tissue cryopreservation (40). The non-membrane-permeable cryoprotectant sucrose was used as a component in vitrification medium at concentrations ranging from 0.5–0.6 M (most commonly 0.5 M) (41). It was reported that the sucrose addition achieved a survival rate of more than 84% of mammalian (mouse, rat, cat, and human) testicular tissue (38, 42).

Combination of the membrane-permeable and non-membrane-permeable media is a commonly used cryopreservation strategy. Previously, the freezing efficiency of the different combinations of 20% GLY, 24% EG, or 20% DMSO with the basic medium [MEM with 0.50 mol/L sucrose and 10% bovine fetal serum (FBS)] was compared for cryopreservation of feline testicular tissue. It was shown that the DMSO/GLY combination showed better maintenance of integrity and the highest percentage of cells capable of proliferation following vitrification (37, 43).

The principle aim while selecting a cryoprotectant is to choose an agent that causes minimal cytotoxicity, including oxidative damage, osmotic damage, cold shock, or chilling injury. The evidence at hand suggests that the effectiveness of a single cryoprotectant is limited, therefore, the better approach is to use them in combination, if we are to maintain the original cellular structure and function. In any case, the use of combination strategy and concentration of cryoprotectants for different freezing methods still needs optimization to achieve best preservation efficiency for genetic resource of felines.

## 4 Transplantation of cryopreserved feline testicular tissue

### 4.1 Testicular tissue transplantation

Testicular tissue transplantation offers the possibility to preserve genetic material and sperm functions in male animals. The first testicular tissue transplantation was performed by John Hunter in 1767 and the rooster testes were successfully transplanted into the abdominal cavity of hens (44). Currently, the autologous transplantation and xenotransplantation are the two main testicular tissue transplantation methods (45). In autologous transplantation, the testicular tissue is removed from and transplanted to the same animal later at the desired time (46). Autologous testicular tissue grafting is useful for studying the production of sperm from cryopreserved prepubertal testicular tissue of human and animals (47). At present, the testicular tissue autologous transplantation has successfully produced sperm and offspring in rhesus (47). Although autologous transplantation of testicular tissue can improve transplant success rates, effectively restore male fertility and reduce the risk of rejection, due to the limits inherent of felines, xenotransplantation seems to be a more practical approach.

In fact, testicular tissue from feline species is more suitable for xenotransplantation (48). In recent years, immature testes from a number of mammals have been used as donors for xenotransplantation, including rodents, pigs, bulls, monkeys, goats, ferrets, iberian lynx (*Lynx pardinus*), cuvier’s gazelle (*Gazella cuvieri*) and mohor gazelle (*Gazel dama mhorr*) (12, 47, 49, 50).

### 4.2 Research progress in testicular tissue transplantation

**Figure 2:**
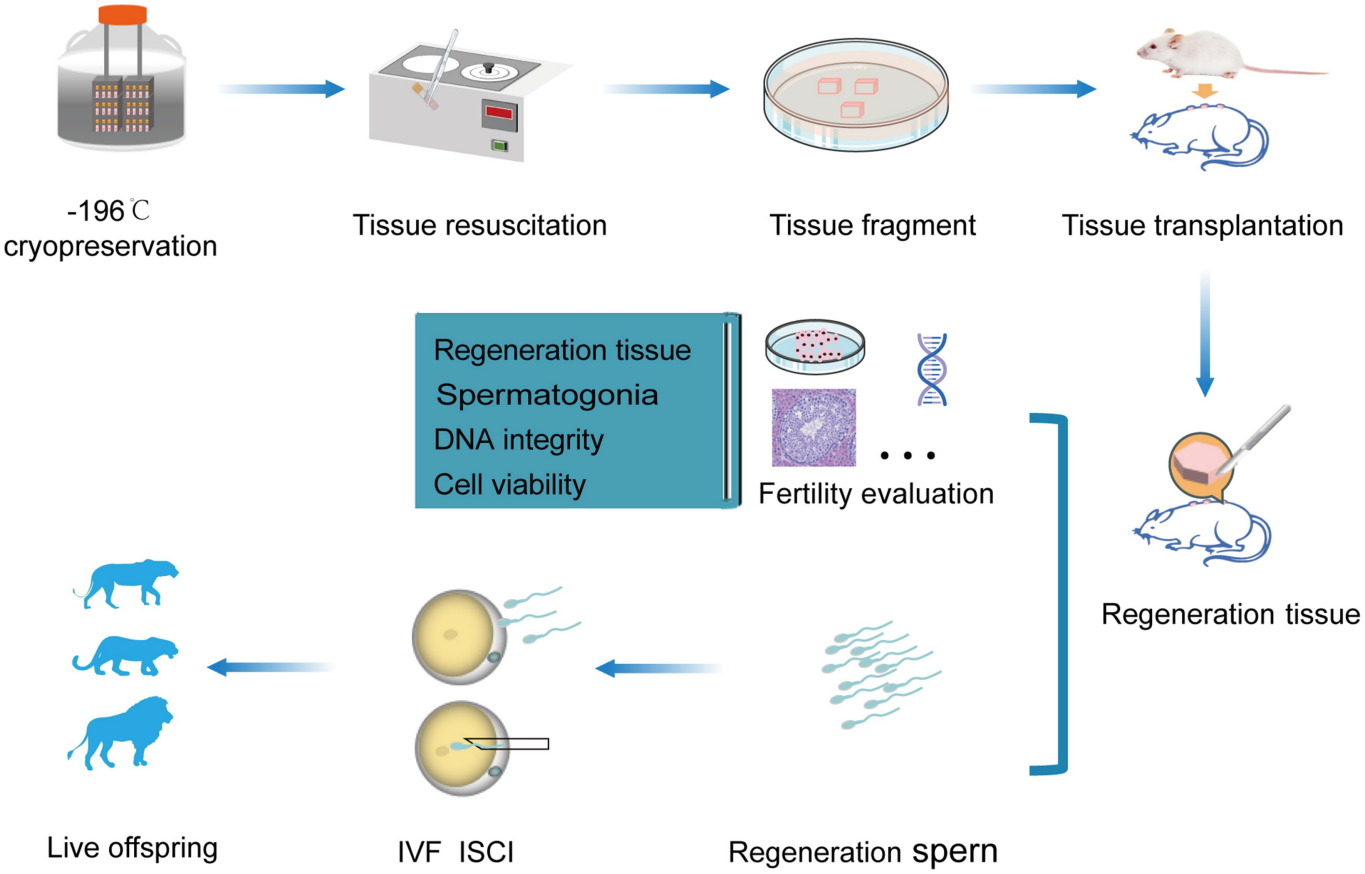
Procedure and utilization of felines testicular tissue transplantation. Transplantation procedure of feline testicular tissue onto the back of immunodeficient mice (above), fertility assessment of regenerated donor sperm after transplantation, and the application of assisted reproductive technologies (below).

The testicular tissue transplantation aims to produce mature donor sperm, and has been tested in mice, hamsters, cats, rabbits, pigs, goats, cows and rhesus monkeys (51). Currently, there are few reports regarding the testicular transplantation in felines. Xenotransplantation of testicular tissue is a technique that several small pieces of tissue are transplanted under the dorsal skin or scrotum of immunodeficient animals. After transplantation, it is necessary to conduct a fertility evaluation of the developed tissue, including assessments of the regenerated tissue, spermatogonia, DNA integrity, and cell viability. When sperm are produced, they can be used for in - vitro fertilization (IVF) and intracytoplasmic sperm injection (ICSI) to generate mature individuals. We guess that the ICSI technique may be a better match for the sperm produced after testicular tissue transplantation because we cannot avoid issues such as low sperm count and potentially poor sperm motility (summarized in Figure 2). Previously, the resuscitated testicular tissue blocks were transplanted under the back skin of 7-12-week-old immunodeficient NCR-nude male mice (12). In another study (14), the influence of age on testicular transplantation in Iberian lynx was investigated (see Table 1 for further information). The formation of seminiferous cords was observed in transplanted testicular tissue from 1–3 days old Iberian lynx, while spermatogonial tubules and germ cells were observed in the testicular tissues transplanted from 6-months-old Iberian lynx. However, when the testicular tissues was transplanted from 2-years-old lynxes, no differentiated germ cells within the tubules were found, but pre-meiotic germ cells were observed. Furthermore, the transplanted testicular tissues from prepubertal Iberian lynx showed spermiogenesis and the percentage of tubules containing sperm gradually increased from 28 to 66 weeks after transplantation. Moreover, sperm proliferation lasts up to 1 year after transplantation with an increased secretion of testosterone. However, no seminiferous tubules and testosterone production were observed in grafts from 2-year-old Iberian lynx, possibly due to the fact that the testicular tissue grafts from mature adult donors might not support germ cell differentiation (14, 52).

Currently, research on testicular tissue transplantation in wild feline species is particularly limited, as available studies are only focused on domestic cats. In shorthaired kittens, it was found that some tubular dilatation occurred around 5 weeks after transplantation, and the germ cells appeared in less than 10% of tubules, and the spermatogonia were seen in the basement membrane of lumen around 14 weeks post-transplantation. Moreover, the round spermatocytes were not found until 26 weeks after transplantation, and elongated spermatocytes were detected firstly at 35 weeks post-transplantation (53). This spermatogenesis continued until 54 weeks after transplantation, suggesting that feline sperm can be recovered from xenografts (53).

The recovery rate of xenografts was highly correlated with the donor age (54). The spermatogonia firstly appeared in 3-weeks-old grafts at 19 weeks and the round spermatocytes appeared at 20 weeks after transplantation. However, the round spermatocytes did not appear in the 5-week-old kitten testis until 26 weeks after transplantation. Moreover, the elongated spermatogonia can be found in the grafts from 5–7 months old donors which is not viable in the 8-month-old xenografts (53). This evidence, although sporadic, shows that the suitable timing for the testicular tissue xenografts is from neonatal to prepubertal age brackets. However, more studies are required to gather more concrete evidence in this regard.

### 4.3 The potential factors influencing the testicular tissue transplantation

Although transplantation of cryopreserved testicular tissue is currently the most promising fertility restoration option, its wide application is hampered by inherent limitations and challenges of tissue cryopreservation and transplantation techniques. The preservation of testicular tissue can be a particularly challenging process due to the complicated tissue structure of testicular tissue (26), as it requires the preservation of various cell types, and maintenance of their three-dimensional structure, and biological activity. Additionally, different types of cells may respond differently to specific cryoprotectants (CPAs) and temperatures (55). Therefore, it may be necessary to design and optimize cryopreservation protocols based on factors such as the age and species of the felines.

The purpose of cryopreservation is to maintain the biological activity of testicular tissue during the long-term storage while minimizing the loss of gametes as much as possible (8). Tissue transplantation is a solid method to test the cryopreservation efficiency and also used to generate sperm for successful fertilization (56). However, the main challenges encountered during testicular tissue transplantation are ischemia and ischemia–reperfusion injury (57). Therefore, efforts are primarily focused on reducing tissue damage caused by inadequate blood supply which is a characteristic of ischemia and ischemia. Enhancing angiogenesis by adding certain substances to reduce ischemic damage and fibrosis, and to maintain the proliferation of spermatogonial stem cells within the testicular tissue after transplantation could be a viable solution. These added substances may include hormones (such as erythropoietin), taurine, and angiogenic growth factors, and others (58).

In summary, strategies aimed at reducing cryopreservation damage to the testicular tissue, increasing early blood supply after transplantation, alleviating post-transplant reproductive cell depletion, and enhancing the antioxidant, anti-apoptotic, and anti-inflammatory capacities of tissues should be the primary focus of future research, if are to improve outcomes of the testicular tissue cryopreservation and transplantation in felines.

## 5 Conclusion and perspective

The cryopreservation and transplantation of testicular tissue are promising techniques in the preservation of genetic diversity and the population of endangered animals including wild felines. As the global biodiversity crisis intensifies due to habitat loss, climate change, and human activities, traditional conservation methods often seem inadequate to address the complex challenges associated with maintaining viable populations of endangered animal species. In this context, testicular tissue cryopreservation and transplantation emerge as viable tools, as these techniques offer significant advantages over traditional conservation methods, allowing for the long-term storage of genetic material with minimal degradation. Unlike traditional methods that rely on the natural reproductive cycles of animals, which can be slow and unpredic, cryopreservation allows for the banking of genetic material that can be used for future breeding efforts, thereby offering greater control and flexibility. Importantly, the donor age, tissue fragment size, cryoprotectants, and freezing procedures are key factors that have influence on cryopreservation efficiency and the subsequent development and sperm regeneration efficiency of the transplanted grafts. Therefore, further refinement and optimization of cryopreservation, transplantation and graft recovery strategies will help to efficiently improve the germplasm conservation effect which could play a crucial role in the conservation of felines and other endangered species in the future.
